# PEDOT-Polyamine-Based Organic Electrochemical Transistors for Monitoring Protein Binding

**DOI:** 10.3390/bios13020288

**Published:** 2023-02-17

**Authors:** Marjorie Montero-Jimenez, Francisco L. Amante, Gonzalo E. Fenoy, Juliana Scotto, Omar Azzaroni, Waldemar A. Marmisolle

**Affiliations:** Instituto de Investigaciones Fisicoquímicas Teóricas y Aplicadas (INIFTA), Departamento de Química, Facultad de Ciencias Exactas, Universidad Nacional de La Plata (UNLP), CONICET. 64 and 113, La Plata B1900, Argentina

**Keywords:** organic electrochemical transistors, conducting polymers, protein binding, PEDOT

## Abstract

The fabrication of efficient organic electrochemical transistors (OECTs)-based biosensors requires the design of biocompatible interfaces for the immobilization of biorecognition elements, as well as the development of robust channel materials to enable the transduction of the biochemical event into a reliable electrical signal. In this work, PEDOT-polyamine blends are shown as versatile organic films that can act as both highly conducting channels of the transistors and non-denaturing platforms for the construction of the biomolecular architectures that operate as sensing surfaces. To achieve this goal, we synthesized and characterized films of PEDOT and polyallylamine hydrochloride (PAH) and employed them as conducting channels in the construction of OECTs. Next, we studied the response of the obtained devices to protein adsorption, using glucose oxidase (GOx) as a model system, through two different strategies: The direct electrostatic adsorption of GOx on the PEDOT-PAH film and the specific recognition of the protein by a lectin attached to the surface. Firstly, we used surface plasmon resonance to monitor the adsorption of the proteins and the stability of the assemblies on PEDOT-PAH films. Then, we monitored the same processes with the OECT showing the capability of the device to perform the detection of the protein binding process in real time. In addition, the sensing mechanisms enabling the monitoring of the adsorption process with the OECTs for the two strategies are discussed.

## 1. Introduction

The development of field effect transistors (FET) has become an actively growing area in the biotechnological field [1,2,3,4,5,6,7,8]. The interest in this technology relies on the simplicity of the instrumentation and the high sensitivity with which label-free and real-time analyte detection can be achieved [9,10]. In particular, organic FETs (oFETs) and organic electrochemical transistors (OECTs) have attracted much attention since they can be easily synthesized with low-cost yielding transistors with high transconductances [11] that can operate in aqueous solutions, facilitating molecule recognition in biological fluids [12]. In this regard, the use of OECTs offers a cost-effective alternative to the surface-sensitive techniques usually employed for the monitoring of biorecognition events and the study of biomolecules interactions, such as surface plasmon resonance (SPR) or biolayer interferometry, which have the disadvantage of requiring expensive and complex instrumentation [13,14,15].

The working principle for sensing with OECTs consists in measuring the changes in the conductivity of the organic channel caused by the variations in the potential difference between the gate electrode and the channel (V_G_). Thus, any change in the charge distribution near the channel surface or the gate electrode leads to a modulation of the V_G_, changing the drain-source current across the polymer (I_DS_). Since in these devices, the gate electrode is immersed in the electrolyte solution, many works have explored the functionalization of different kinds of gate electrodes for the detection of proteins and a variety of metabolites [16,17].

However, a very interesting feature of OECTs is that the polymeric channel can be functionalized to perform direct detection on its surface. In fact, conducting polymers are ideal materials for this purpose as their synthesis can be tuned to obtain channels with different compositions, allowing controlling their physicochemical features, such as pH sensitivity, conductivity, redox switching potential range, and endow the surface with functional groups that can be used for the immobilization of biorecognition elements or other moieties of interest [18].

Among the conductive polymers that have been used as OECT channels for biosensing [19], PEDOT is the most employed one since it yields devices possessing biocompatible environments, high transconductances values, and requiring low operational voltages (less than 1 V) [20]. Disadvantages of using PEDOT are related to its poor capability of chemical functionalization and low ionic charge that hindered the electrostatic interaction with charged molecules. Particularly, the electrochemical features of PEDOT improve when it is combined with doping agents, such as tosylate and other organic anions [21,22,23,24]. In particular, a very interesting material for the construction of OECTs is obtained from the combination of PEDOT with polyallylamine hydrochloride (PAH) [25]. In this regard, PEDOT-PAH blends have been recently shown to maintain the high electroactivity of bare PEDOT films, with the advantage of exhibiting amine groups that can be used for sensing and biosensing through different mechanisms. In the first place, the amino moieties on the polymer matrix confer positive charges to the surface, allowing the electrostatic interaction with negatively charged entities [25]. This is interesting since many biomolecules are negatively charged at physiological pH [26]. On the other hand, these groups also can be used to covalently modify the surface, allowing the anchoring of recognition elements, such as proteins or antibodies [27]. Moreover, the presence of the amino groups in the material increases the pH sensitivity of the channel surface, which can be used for the monitoring of biochemical processes that generate pH changes [28,29]. For instance, acetylcholinesterase has been incorporated into PEDOT-PAH films for the detection of the neurotransmitter acetylcholine [25]. It has been shown that the hydrolysis of the substrate catalyzed by the enzyme attached to the channel generates a decrease in the local pH that can be monitored by the OECTs.

In this work, we explore the use of PEDOT-PAH-based OECTs for studying protein binding events. Firstly, we use surface plasmon resonance (SPR) to study the integration of proteins to PEDOT-PAH films through different functionalization strategies: (i) The direct deposition of Glucose Oxidase (GOx), chosen as a model protein to evaluate the biomolecule binding through electrostatic interactions with the PEDOT-PAH film and (ii) the covalent attachment of mannose to the surface to build a Concanavalin A (ConA)-GOx assembly [30,31,32], in order to evaluate the protein incorporation through a biorecognition process. Then, we monitored these functionalization processes with the OECT configuration to show the potential of these transistors for the detection of protein binding events in real time. Finally, the sensing mechanisms involved in the monitoring of the macromolecule adsorption with the OECT in the two strategies are discussed.

## 2. Materials and Methods

### 2.1. Reagents

For the experiment, 3,4-ethylenedioxythiophene (EDOT) (Lot#WXBC1309V, Wuxi, China), PAH (M_w_ ~ 58 kDa, Lot# MKBW4380V, St. Louis, MO, USA), poly (sodium 4-styrenesulfonate) (PSS) (M_w_ ~ 70.000), divinylsulfone (DVS) and ConA (from Canavalia ensiformis (Jack Bean): Lot#097K7670, St. Louis, MO, USA catalog number: C2010) were purchased from Sigma Aldrich. Pyridine (99%) was obtained from Biopack and Fe (III) p-toluenesulfonate (Fe (III) Tos) (38–42% in n-butanol) was obtained from Heraeus. N-butanol (99.4%), KCl, Na_2_CO_3,_ and HEPES were purchased from Anedra (Los Troncos del Talar, Buenos Aires, Argentina), and glucose oxidase (GOx) was purchased from Calzyme (from Aspergillus Niger: Lote#67-8-18 As, Tulelake, CA, USA).

### 2.2. PEDOT-PAH Films Synthesis

PEDOT-PAH films were chemically synthesized on Au substrates (SPR102, BioNavis) (to be employed in the mass density determinations) and on interdigitated electrodes (Micrux ED-IDE1-Au, 10/10 µm electrode/gap) (for the construction of the OECTs) according to a previously reported protocol [33]. Before the synthesis, the substrates were cleaned with basic piranha (NH_4_OH 35% and H_2_O_2_ 30% 1:1) and ethanol. Then, an oxidant solution was prepared by mixing 715 μL of the Fe (III) Tos solution, 4 μL of butanol and 16.5 μL of pyridine. Next, 200 μL of a PAH solution (40 mg PAH/200 μL water) was incorporated, and the obtained solution was mixed with 12.5 μL of the EDOT monomer, homogenized in a vortex, and filtered (pore diameter = 0.2 µm). Immediately, the resultant mixture was deposited on the interdigitated electrodes by spin-coating employing a rotation rate of 1000 rpm for 1 min and an acceleration of 500 rpm s^−1^. Then, the electrodes were heated at 70 °C to favor the polymerization. During this step, EDOT is chemically oxidized by Fe (III) ions to the produced PEDOT [34,35]. Finally, electrodes were washed with water and dried.

The mannosylation of PEDOT-PAH films was performed as previously reported [27]. Briefly, the PEDOT-PAH substrate was incubated in a 5% DVS in Na_2_CO_3_ pH = 11 solution for 1 h and rinsed with Na_2_CO_3_ solution. Then, the mannosylation was carried out by immersing the substrate in a 10% mannose solution in Na_2_CO_3_ pH = 11 for 18 h and rinsed with Tris buffer (Tris 10 × 10^−3^ M, NaCl 0.1 M, and pH = 7.5).

### 2.3. Raman Spectroscopy

PEDOT and PEDOT-PAH films were synthesized on glass substrates, following the procedure described above for the OECTs fabrication, and their Raman spectra were acquired. Three different PEDOT-PAH films were prepared by increasing the amount of PAH in the polymerization mixture (15 mg PAH for PEDOT-PAH 1, 40 mg for PEDOT-PAH 2, and 75 mg for PEDOT-PAH 3 in 200 µL Milli-Q water). An i-Raman BW415-532S (BWTek) Raman Spectrometer was employed, using a laser wavelength of 532 nm and a power of 248 mW, that was focused with a 20× optical microscope (BAC151B, BWTek). Each sample was measured in four different spots. The spectral region analyzed ranged from 0 to 4000 cm^−1^.

### 2.4. SPR Measurements

A multi-parametric surface plasmon resonance (MP-SPR) instrument SPR Navi 210 A (BioNavis Ltd., Tampere, Finland) was employed to monitor the adsorption of different macromolecules on the PEDOT-PAH modified gold substrates. Lasers of 670 nm and 785 nm were employed. For this purpose, PEDOT-PAH films were deposited on the Au SPR substrates (BioNavis Ltd.) using the above-mentioned protocol. For the monitoring of PSS adsorption on the modified substrate, a 0.1 mg/mL in 0.1 M KCl solution was injected under a flow rate of 15 μL/min, while the SPR angular scans were recorded. After signal stabilization, a rinsing step was carried out with KCl. For GOx electrostatic adsorption, the same procedure was performed employing a 1 mg/mL GOx in 10 mM KCl and 1 mM HEPES pH = 7.2 buffer solution and rinsing with buffer. For the study of ConA and GOx recognition, the ex-situ mannosylation of the surface was first performed. Then the modified substrate was placed on the SPR cell, and a 10 μM ConA solution in 10 mM KCl and 1 mM HEPES pH = 7.2 buffer was injected and then rinsed with buffer. The same procedure was performed to study the GOx binding on the ConA-modified surface employing a 30 μM GOx in buffer solution.

### 2.5. Electrochemical Measurements

The transfer curves were obtained by applying a drain-source voltage (V_DS_) of −50 mV and registering the drain-source current (I_DS_) as a function of the gate potential (V_G_) after and before each modification step at a scan rate of 10 mV s^−1^. For the study of the PSS adsorption the transfer curves (I_DS_ vs. V_G_) were measured in 0.1 M KCl, whereas for the monitoring of the protein adsorption, transfer curves were measured in 10 mM KCl and 1 mM HEPES buffer. The real time monitoring of each adsorption process was obtained by registering the I_DS_ while injecting the solution containing the macromolecule at constant V_G_ and a flow rate of 50 µL min^−1^. The V_G_ value was chosen from the transfer curve of the transistor on the modification step prior to the adsorption process under study, as the gate potential at which the I_DS_ reaches 60% of the maximum current. It was considered that this potential is the lowest value at which the transconductance is high enough to obtain a good detection sensitivity. All electrochemical measurements were carried out employing a TEQ bipotentiostat and a Micrux Technologies flow cell with a wire Ag/AgCl electrode as gate.

## 3. Results and Discussion

### 3.1. PEDOT-PAH Film Preparation and Characterization

**Figure 1:**
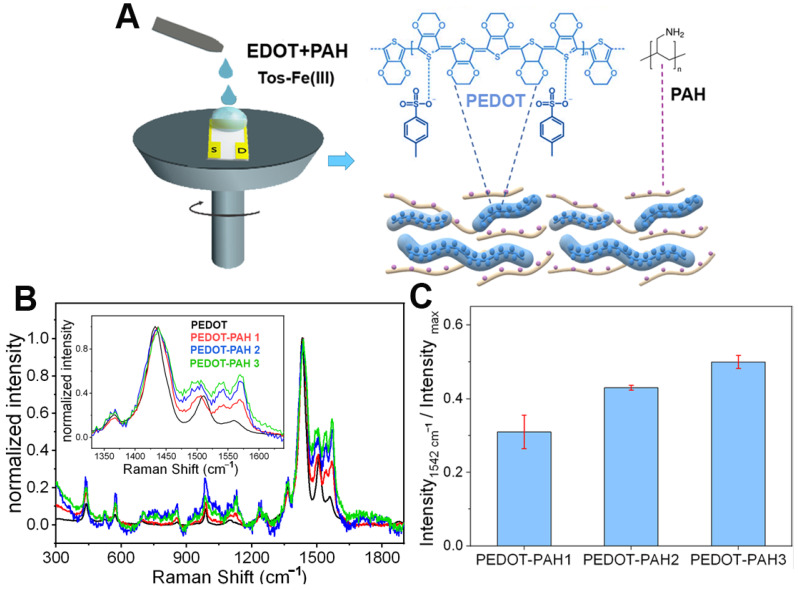
(**A**) Scheme of the synthesis of PEDOT-PAH films by spin coating. (**B**) Raman spectra of PEDOT (black), PEDOT-PAH 1 (red), PEDOT-PAH 2 (blue), and PEDOT-PAH 3 (green) films deposited on glass substrates. (**C**) Relative intensity of the Raman peak at 1541 cm^−1^ to the maximum intensity (Intensity _max_), corresponding to the peak at 1431 cm^−1^, for PEDOT-PAH blends of different compositions. Error bars correspond to SD (*n* = 4).

PEDOT-PAH-based OECTs were prepared by chemical polymerization. To this end, the synthesis mixture containing the monomer (EDOT) and the PAH in a butanol/water medium was deposited on interdigitated substrates by spin coating (Figure 1A). The integration of PAH to the PEDOT matrix was corroborated by Raman spectroscopy. In Figure 1B, representative Raman spectra of pristine PEDOT and the PEDOT-PAH blends with increasing PAH proportions (normalized by the value of maximum Raman intensity for the sake of comparison) are shown. In all the samples, the characteristic bands of PEDOT reported in the literature can be observed [36,37]: The peak at 439 cm^−1^ that is assigned to SO_2_ bending, the peaks at 570 and 989 cm^−1^ that are related to oxyethylene ring deformation, and the peaks at 1241, 1362, 1431, and 1510/1563 cm^−1^ that correspond to C_α_–C_α_ inter-ring stretching, C_β_–C_β_ stretching, symmetric C_α_ = C_β_(−O) stretching, and C_α_ = C_β_ stretching, respectively (Figure 1B). In addition, for the PEDOT-PAH blends, a peak at 1542 cm^−1^ is also observed, which has been attributed in the literature to bending modes of amine groups or to changes in the doping state of PEDOT [27,38,39]. Moreover, the intensity of this peak increases with the PAH concentration in the synthesis solution, confirming the integration of the polyallylamine into the polymer matrix. In Figure 1C, the ratios of the Raman intensity value at 1542 cm^−1^ to the value at the peak of maximum intensity (at 1431 cm^−1^) are shown. Spectra acquired on different spots on the same sample are closely similar. Bars in Figure 1C indicate the SD of the intensity ratio corresponding to four different positions on the same sample.

**Figure 2:**
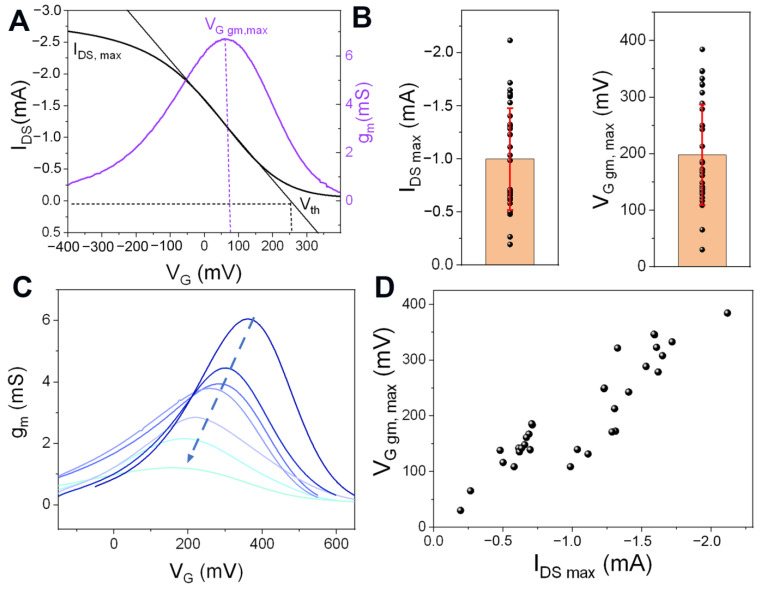
(**A**) Characteristic transfer curve and transconductance as a function of the gate potential for a PEDOT-PAH-based OECT. (**B**) I_DS max_ and V_G gm, max_ for 30 different OECTs synthesized in the same conditions. Bars represent the mean values, and the error bars are the SD. (**C**) g_m_ vs. V_G_ for seven representative OECTs (arrow indicates decreasing transconductance values corresponding to thinner films). (**D**) V_G gm, max_ as a function of I_DS max_ for different PEDOT-PAH-based OECTs. (KCl 10 mM + HEPES 1 mM, V_DS_ = −50 mV).

As was shown elsewhere [25], the optimization of the channel synthesis is an important step in obtaining efficient sensors. In particular, the composition and the thickness of the polymer film that acts as a channel between the drain and source terminals not only affect the current values of the transistors but also the gate potential range in which the redox commutation occurs. This is relevant since it is in this region where the transistor shows the highest transconductance (g_m_ = dI_DS_/dV_G_) and therefore, the highest sensitivity to the modulation of the electric field upon biding events. Thus, the OECTs parameters defining the operational gate potential region (i.e., threshold voltage, V_th_, and maximum transconductance voltage, V_G gm, max_ (see Figure 2A)) depend on the synthesis conditions. For instance, increasing the PAH/EDOT ratio in the synthesis solution decreases the V_th_ and the conductivity of the material [25]. In addition, employing more diluted synthesis solutions or increasing the rotation rate of the spin coating allows to a decrease in the thickness of the films [40], which yields a decrease of the maximum current (I_DS,max_) and the V_G gm, max_ of the transistors [33,41]. Then, when fabricating OECTs for biosensing purposes, these characteristic parameters of the transistor response can be controlled to avoid the use of high voltages that may cause the deterioration of the transistors due to undesirable reactions or affect biomolecules attached to the surface. In this work, the synthesis conditions (see experimental section) were chosen based on a protocol previously designed for the optimization of PEDOT-PAH films as OECTs channels that provides films with high transconductances at low gate potential values (V_G gm, max_ values between 0 and 300 mV) [33]. The transfer curves of the films obtained with this procedure were measured in buffer solution and the characteristic parameters, I_DS max_ and V_G gm max_, are shown in Figure 2B. A dispersion in both parameter values for the different transistors synthesized in the same conditions can be observed. The dispersion in I_DS max_ could be ascribed to variations in the thickness of the films within the reproducibility limits of the synthesis method since lower I_DS max_ values are expected for thinner films. To corroborate this hypothesis, 19 silicon substrates were modified with PEDOT-PAH films in the same conditions, and the thicknesses of the films were estimated employing ellipsometry (See Supplementary Information). A similar dispersion of the thickness values to that of I_DS max_ was obtained (the SD of the thickness values represents 44% of the thickness mean value, while the SD of the I_DS max_ values represents 48% of the I_DS max_ mean value (see Supplementary Information)). In addition, in Figure 2C, the transconductance as a function of V_G_ is shown for seven representative OECTs. It can be observed that, as the maximum current and the transconductance of the films decreases, the V_G gm, max_ values shift to lower gate potentials, which explains the dispersion on the maximum transconductance voltage values. The correlation between the V_G gm, max,_ and the maximum I_DS, max_ current is shown in Figure 2D.

### 3.2. Protein Sensing Based on Electrostatic Interactions

**Figure 3:**
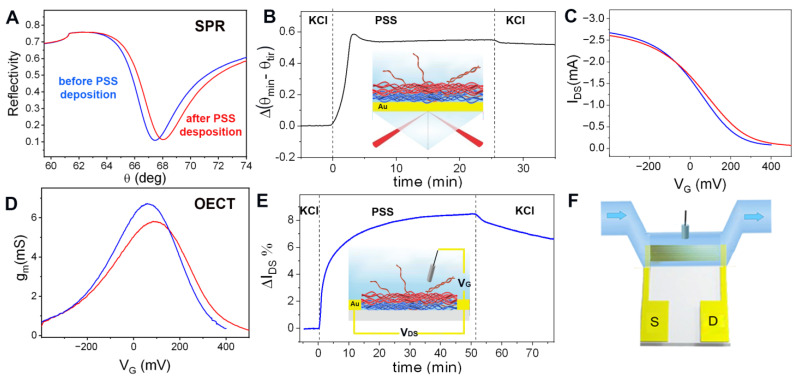
(**A**) Reflectivity curves of the PEDOT-PAH substrate before (blue) and after (red) PSS deposition. (**B**) Real-time change in the SPR signal Δ(θ_min_–θ_tir_) during the deposition of PSS on a gold substrate modified with PEDOT-PAH under flow conditions. (**C**) Transfer curves and (**D**) transconductance values of a PEDOT-PAH OECT in 0.1 M KCl before (blue) and after (red) PSS deposition (**E**) Real time change in the OECT ΔI_DS_% current during PSS adsorption. (**F**) Scheme of the flow cell employed for the electrochemical measurements.

Recently, the capability of PEDOT-PAH-based OECTs for sensing the deposition of charged macromolecules on the channel surface has been shown, allowing the real time monitoring of the assembly of a polyelectrolyte multilayer by Layer-by-Layer (LbL) technique [33]. It was observed that the adsorption of negatively charged macromolecules generates an increase in the material conductivity due to an increase in the hole carrier concentration, shifting the transfer characteristic curves to more positive V_G_ values. Here, we explore the capability of using the electrostatic interaction of the PEDOT-PAH channel with charged macromolecules to monitor protein binding. To this end, we employed SPR to monitor the adsorption of macromolecules and then correlate this signal to the OECT response. Firstly, we performed the comparative study employing PSS as a model of a negatively charged macromolecule since it has been proved that it can be electrostatically deposited on the PEDOT-PAH surface generating a change in the OECT current signal [33]. In Figure 3A, the changes in the minimum reflectance angle (θ_min_) during the injection of a 0.1 mg/mL solution of PSS in 0.1 M KCl are shown. To eliminate the variations of bulk refractive index changes, the total internal reflection angle change (θ_tir_) was subtracted from θ_min_. After injection, a rapid increase in the signal is observed, followed by a small decrease when rinsing with KCl solution that can be attributed to the desorption of weakly adsorbed PSS chains [42]. From the change in the SPR signal before and after PSS injection, the polyelectrolyte mass density deposited was estimated to be 535 ng cm^−2^ (see Figure 3A,B and Supplementary Information). Next, we monitored the same adsorption process using the PEDOT-PAH OECT. To this end, the transfer curves in 0.1 M KCl were measured before and after PSS deposition (Figure 3C,D). A shift of the transfer curve to more positive gate potentials is observed, demonstrating the stabilizing effect of the hole carriers on the polymer matrix by adsorption of negative charges on the channel surface. In addition, the real time response of the OECT at a constant gate voltage was recorded while injecting the PSS solution under flow conditions (the same conditions as those employed for the SPR measurements) (see the scheme of the setup in Figure 3F). In Figure 3E, the relative changes in the drain-source current are shown in terms of I_DS_% = 100 (I_DS_-I_0_)/I_0_, where I_0_ is the current before the injection measured at V_G_ = 100 mV. An increase in the OECT current signal is observed in agreement with the shift of the transfer curve to more positive potentials. However, the time required to observe the deposition process with the OECT is higher than the time required to observe the same process with SPR. Whereas the SPR response stabilizes in less than 7 min, the OECTs need more than 40 min to reach a plateau. This effect has been previously observed for the deposition of the polyanion on the PEDOT-PAH film [33] and can be explained by considering that the SPR signal is only influenced by the mass density on the surface while the current measured with the OECT also depends on the ion concentration inside the polymer channel. The interdiffusion of PSS chains into the PEDOT-PAH compensates positive charges in the film, generating a reorganization of the film chains that is accompanied by the release of counterions. This ion flux through the polymer generates a capacitive contribution on the OECT current, characterized by a slow kinetic compared to the mass deposition process [43,44].

In order to extend this strategy to biochemical systems, we carried out the same procedure to study the adsorption of negatively charged proteins using GOx as a model system. The immobilization of proteins through electrostatic interactions represents an interesting modification strategy since it provides an easy method for attachment as it diminishes the probability of altering its functionality compared with procedures that involve covalent reactions [45]. In addition, GOx has been previously used as a probe for studying interactions with positively charged surfaces [46] as it has a negative net charge at physiological conditions (isoelectric point ≈ 4.2) [47]. Thus, we were able to perform the direct electrostatic adsorption of the enzyme harnessing the positive charges of the PEDOT-PAH surface.

**Figure 4:**
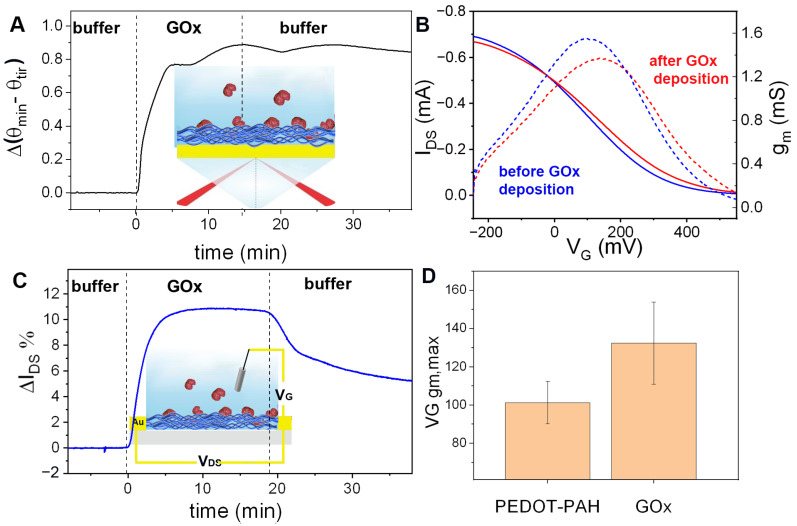
(**A**) Real time change in the SPR signal Δ(θ_min_–θ_tir_) during GOx deposition on a gold substrate modified with PEDOT-PAH under flow conditions. (**B**) Transfer curves and g_m_ vs. V_G_ of a PEDOT-PAH OECT in 10 mM KCl and 1 mM HEPES pH = 7.2 before (blue) and after (red) GOx deposition. (**C**) Relative change in the OECT I_DS_ current during GOx adsorption. (**D**) V_G gm, max_ changes (average of four devices. Error bars correspond to SD). V_DS_ = −50 mV and V_G_ = 54 mV.

The SPR response to the injection of 1 mg/mL GOx in 10 mM KCl and 1 mM HEPES (pH = 7.2) solution was measured under continuous flow conditions (Figure 4A). From the increase in the SPR signal, a mass density of 505 ng cm^−2^ was estimated (see Supplementary Information). In addition, it can be observed that the Δ(θ_min_–θ_tir_) signal does not change after rinsing with buffer solution, showing the stability of the assembly. Next, the deposition of GOx on PEDOT-PAH OECTs was studied by monitoring the transistor response to the injection of the same GOx solution as that employed for the SPR measurements (Figure 4B,C). In Figure 4B, the transfer curve and the transconductance as a function of V_G_ are shown for an OECT before and after GOx adsorption. A shift of the curve to higher gate potential values was observed after GOx deposition, similar to the effect observed for PSS adsorption (Figure 3C). In Figure 4D, the obtained shift of V_G gm, max_ is shown. This interaction was further studied in real time measurements by using a flow condition. As observed in Figure 4C, there is an increment in I_DS_ caused by the exposition of the transistor channel to the GOx solution, which could be ascribed to the interaction of the negative protein with the positively charged PEDOT-PAH film. This increase in the OECT signal could be addressed by the stabilization of the hole carriers in the PEDOT-PAH channel generated by the negative charges of the enzyme. Note that after the injection of the protein, a decrease in the current is observed during the injection of buffer solution. As can be seen in the SPR measurements, no desorption of the GOx takes place after rinsing. Therefore, the change in the current observed when passing the GOx solution has another contribution besides the one related to the protein adsorption on the surface. This extra contribution may be related to changes in the ionic strength of the solution due to the presence of the charged enzyme or weak adsorption of the protein on the gate electrode that is desorbed after rinsing with buffer. However, a net increase in the current value is obtained after rising with buffer compared with the initial value before the injection of GOx. The same procedure was performed in four devices, and in all of them, an increase in the current was observed upon GOx adsorption.

### 3.3. Protein Sensing Based on Specific Biorecognition

Once determined the capability of the PEDOT-PAH OECTs for the detection of protein electrostatic adsorption, we proceeded to explore the device response to protein binding driven by specific recognition events. To this end, the glycosylation of the polymer film was carried out, in order to study the specific binding of a lectin (Concanavalin A, ConA) [27]. ConA is usually employed as a binder protein to integrate functional glycoproteins, such as active building blocks. Then, we studied both the recognition of ConA by a glycosylated-PEDOT channel and the subsequent interaction of bound ConA with other glycoenzyme by monitoring the changes in the OECTs response. Being GOx also a glycoprotein, it was further used as a model for studying the specific interaction with surface-bound ConA.

Although GOx effectively adsorbs on PEDOT-PAH surface (mainly by nonspecific electrostatic interactions between the negatively charged enzyme and the positively charged surface amino groups of PAH), the mannosylation of the surface has been proved to prevent this nonspecific binding [48]. Contrarily, after mannosylation, the binding of the lectin ConA is possible as it takes place by specific biorecognition interactions (lectin-carbohydrate binding). Furthermore, surface-bound ConA has been shown to be able to bind glycosylated proteins (as GOx and HRP), allowing for the construction of functional PEDOT-PAH-mannose-ConA-GOx interfaces [48]).

**Figure 5:**
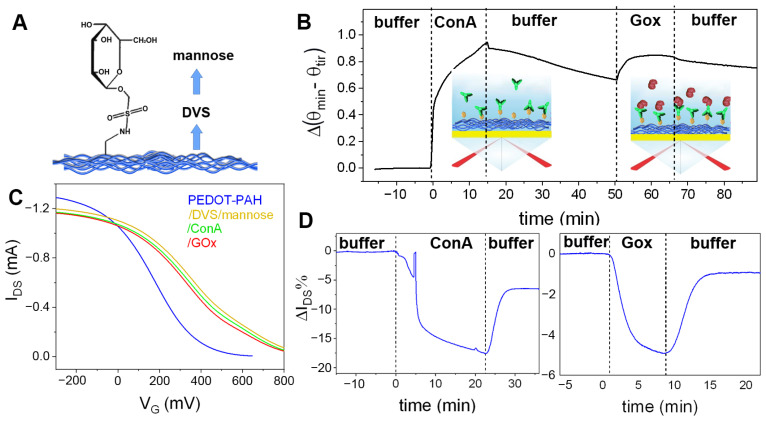
(**A**) Scheme of the PEDOT-PAH films covalently modified with DVS and mannose employed for the study of specific recognition of ConA. (**B**) Change in the SPR signal Δ(θ_min_–θ_tir_) during the subsequent deposition of ConA and GOx on the gold substrate modified with PEDOT-PAH, DVS, and mannose under flow conditions. (**C**) Transfer curves of a PEDOT-PAH OECT before (blue) and after DVS-mannose (yellow), ConA (green), and GOx (red) modification steps. (**D**) Real time changes in the I_DS_% upon ConA and GOx binding (10 mM KCl + 1 mM HEPES, pH = 7.2).

As a first step, the covalent anchoring of mannose was performed on the PEDOT-PAH film employing DVS as a linker between the amino groups of the conducting channel and the -OH groups of the carbohydrate (Figure 5A) [27]. Then, the binding of ConA on the mannosylated surface, using the specific biorecognition interactions between the lectin and surface mannose residues, was studied by SPR and OECTs comparatively. To this end, a gold substrate previously modified with PEDTOT-PAH, DVS, and mannose was employed for the monitoring of ConA anchoring on the modified conducting polymer film by SPR. Moreover, after rinsing with buffer solution, the subsequent injection of GOx solution was carried out to monitor the recognition of the glycoprotein by the carbohydrate-binding sites of ConA. In Figure 5B, the consecutive increases in the SPR signal Δ(θ_min_–θ_tir_) are shown. From the total SPR signal change, the mass densities corresponding to each deposition resulted to be 0.404 and 0.062 μg cm^−2^ for ConA and GOx, respectively (see Supplementary Information).

Next, the same system was studied by the OECT using mannosylated PEDOT-PAH films as conducting channels. In Figure 5C, it is shown that the modification of the surface channel with DVS and mannose generates a markedly positive shift of the OECT transfer curve. This shift to positive gate voltages can be explained as a consequence of the reaction of the amino groups, revoking the de-doping effect of the PAH in the PEDOT channel [33]. Next, the modified OECT was employed to monitor the real time biorecognition of ConA. After the injection of a 10 μM solution of ConA a decrease in the I_DS_ is observed, as shown in Figure 5D. Note that ConA is negatively charged at neutral pH (the isoelectric point is between 4.5 and 5.5) [49], so an increase in the current would be expected if only electrostatic interaction was considered, following the behavior shown before for the electrostatic adsorption of PSS and GOx. However, the opposite effect is observed here. Moreover, the transfer curve of the OECT shifts to the opposite direction of that observed with the electrostatic adsorption of the negatively charged macromolecules (Figure 5C). In addition, the subsequent injection of GOx to the surface also generates a decrease in the current and a shift of the transfer to lower V_G_ values. This experiment was repeated in four devices, observing decreases of the current upon ConA and GOx binding in all of them (see Supplementary Information). The nature of the interaction causing the decrease in the registered current will be discussed in the next section.

### 3.4. Sensing Mechanisms for Monitoring Protein Binding on PEDOT-PAH-Based OECTs

Field-effect transistors (FETs) have been widely employed for biosensing purposes enabling the detection of many analytes going from ions and small molecules to proteins, DNA, and virus particles [17,50,51,52,53]. In this regard, a variety of sensing strategies have been explored to perform analyte detection in different experimental conditions. Among them, sensing the direct electrostatic interactions between binding charged analytes and the surface [54] or the changes in the interface impedance upon the biomolecule adsorption [55] and the construction of dense coating layers atop the surface to enable detection through Donnan mechanisms [56,57] are the most employed ones. In general, the capability of FETs to sense through one or another mechanism depends on the characteristics of the electrolyte solution (being the ionic strength the most important parameter) [58], the charge of the analyte, and the properties of the coating layer that contains the binding groups or recognition elements [59,60]. However, in the case of organic FETs and OECTs, there is another relevant parameter affecting the sensing mechanism, which is the chemical composition of the channel. A clear example of the importance of the channel nature on the detection mechanism arises from the comparison of the sensing response of two PEDOT-based OECTs doped with different polyelectrolytes to the construction of polyelectrolytes multilayers atop the channel. In the first case, Pappa et al. performed the monitoring of the construction of poly-L-lysine (PLL) and PSS assembly by LbL technique atop a PEDOT:PSS channel [61]. They observed that the subsequent adsorption of positively charged PLL and negatively charged PSS generated a decrease in the I_DS_ independently of the charge of the pendant groups in the outermost layer. While a decrease in the current was expected for PLL adsorption due to the charge compensation of the PSS chains that stabilized the hole carriers in the PEDOT matrix, the opposite effect would be expected for the subsequent PSS adsorption. However, a continuous decrease in the current was observed for all the layers. The authors assigned this behavior to an additional capacitance that is added with each polyelectrolyte layer and proposed that the deposition of successive polymer layers generates a less effective gating on the channel. Oppositely, a similar experiment performed on a PEDOT-PAH-based OECT showed that the adsorption of polyelectrolytes generated alternating decreases or increases in the current following the sign of the charge of the polyelectrolyte at the outermost layer [33]. Whereas the adsorption of positively charged poly-(diallyldimethylammonium) chloride (PDADMAC) yielded a decrease in the device signal, the adsorption of PSS generated an increase in the current. This indicates that when PAH is de-doping the PEDOT matrix, it is possible to sense the electrostatic interaction of charged macromolecules adsorbed on the surface. This explains the shift to higher gate values and the increase in the film conductivity that was observed when negatively charged PSS or GOx were deposited on the PEDOT-PAH surface shown in this work (see Figure 3 and Figure 4). Here, it is important to notice that the presence of PAH in the PEDOT-based channel markedly shifts the V_G g, max_ and V_G,th_ to lower values compared to pristine PEDOT, indicating an effective de-doping of the conductive polymer. Thus, the adsorption of negatively charged moieties on the surface compensates for the positive charges of the PAH, shifting the gate potential towards the characteristic gate potential values of pristine PEDOT. Oppositely, in the case of the DVS-modified polymer, this mechanism is not enabled since the neutralization of the positive charge that originally provided the amino groups of PAH suppresses the electrostatic effect caused by further adsorption of charged proteins, such as ConA or GOx. In fact, a decrease in the current is observed upon the adsorption of the two proteins on the DVS/mannose-modified surface, suggesting that a different sensing mechanism operates when the amino groups are not available to interact with negatively charged entities.

It is well known that, in OECTs, the charge transport has an electronic contribution, given by the intrinsic charge carriers within the polymer, and an ionic contribution, arising from the ion flux between the electrolyte solution and the organic film to maintain charge balance in the channel [62]. Then, for mixed ionic-electronic conductors such as PEDOT, a capacitance is generated on the polymer/electrolyte interface (as well as at the gate electrode/electrolyte solution interface). It has been reported for a PEDOT-based OECT that the capacitance associated with the deposition of a macromolecule layer atop the channel yields a decrease in the device output current [61]. Thus, the decrease in I_DS_ observed upon ConA adsorption on the mannosylated PEDOT-PAH surface can be ascribed to changes in the capacitance at the polymer film/solution interface. We hypothesize that the presence of positive charges de-doping the PEDOT matrix favors the direct electrostatic detection mechanism, enabling the device to sense the modulations of the electric field in the vicinities of the surface upon the adsorption of charged macromolecules. On the contrary, if the positive groups of the polymer are used for the covalent attachment of recognition elements, the detection through a direct electrostatic mechanism is disadvantaged. Still, the recognition event can be monitored based on the changes in the impedance of the channel/solution interface upon the binding process.

## 4. Conclusions

PEDOT-PAH films have been prepared, characterized, and employed as conducting channels to monitor protein binding using glucose oxidase as a model system. Two strategies have been explored: The direct electrostatic adsorption of GOx and the mannosylation of the PEDOT-PAH surface to perform the specific recognition of ConA and the subsequent GOx binding. The modification steps have been corroborated by SPR measurements showing the formation of stable assemblies by the two strategies. Then, the binding processes were successfully monitored by the OECT. It was demonstrated that the direct adsorption of GOx on the PEDOT-PAH channel can be monitored in real time by measuring the I_DS_ changes in the potential region of high transconductance thorough an electrostatic mechanism. For the second strategy, the covalent modification of the PEDOT-PAH surface by reaction of the amino groups with DVS and mannose was performed, decreasing the proportion of positively charged amino groups. This modification would suppress the electrostatic mechanism for sensing the adsorbed charges on the surface. The decrease in the polymer channel conductivity upon protein binding observed in this case can be explained by the changes in the channel/solution interface impedance. The results presented here not only show the potential of PEDOT-PAH-based OECTs for protein adsorption monitoring and the construction of biosensing architectures but also give new insight into the sensing mechanisms with PEDOT-based devices.

## Data Availability

The data presented in this study are available on request from the corresponding authors.

## Supplementary Materials

The following supporting information can be downloaded at: https://www.mdpi.com/article/10.3390/bios13020288/s1, Figure S1: Thickness of PEDOT-PAH films prepared on Si substrates; Figure S2: Shifts in V_G gm, max_ upon functionalization.

## References

[1] Vu C.A., Chen W.Y. (2019). Field-Effect Transistor Biosensors for Biomedical Applications: Recent Advances and Future Prospects. Sensors.

[2] Chen H., Zhang W., Li M., He G., Guo X. (2020). Interface Engineering in Organic Field-Effect Transistors: Principles, Applications, and Perspectives. Chem. Rev..

[3] Sadighbayan D., Hasanzadeh M., Ghafar-Zadeh E. (2020). Biosensing Based on Field-Effect Transistors (FET): Recent Progress and Challenges. TrAC Trends Anal. Chem..

[4] Wang S., Hossain M.Z., Shinozuka K., Shimizu N., Kitada S., Suzuki T., Ichige R., Kuwana A., Kobayashi H. (2020). Graphene Field-Effect Transistor Biosensor for Detection of Biotin with Ultrahigh Sensitivity and Specificity. Biosens. Bioelectron..

[5] Macchia E., Torricelli F., Bollella P., Sarcina L., Tricase A., Di Franco C., Österbacka R., Kovács-Vajna Z.M., Scamarcio G., Torsi L. (2022). Large-Area Interfaces for Single-Molecule Label-Free Bioelectronic Detection. Chem. Rev..

[6] Macchia E., Manoli K., Di Franco C., Picca R.A., Österbacka R., Palazzo G., Torricelli F., Scamarcio G., Torsi L. (2020). Organic Field-Effect Transistor Platform for Label-Free, Single-Molecule Detection of Genomic Biomarkers. ACS Sens..

[7] Picca R.A., Manoli K., Macchia E., Sarcina L., Di Franco C., Cioffi N., Blasi D., Österbacka R., Torricelli F., Scamarcio G. (2020). Ultimately Sensitive Organic Bioelectronic Transistor Sensors by Materials and Device Structure Design. Adv. Funct. Mater..

[8] Rivnay J., Inal S., Salleo A., Owens R.M., Berggren M., Malliaras G.G. (2018). Organic Electrochemical Transistors. Nat. Rev. Mater..

[9] Berninger T., Bliem C., Piccinini E., Azzaroni O., Knoll W. (2018). Cascading Reaction of Arginase and Urease on a Graphene-Based FET for Ultrasensitive, Real-Time Detection of Arginine. Biosens. Bioelectron..

[10] Xu S., Jiang S., Zhang C., Yue W., Zou Y., Wang G., Liu H., Zhang X., Li M., Zhu Z. (2018). Ultrasensitive Label-Free Detection of DNA Hybridization by Sapphire-Based Graphene Field-Effect Transistor Biosensor. Appl. Surf. Sci..

[11] Inal S., Malliaras G.G., Rivnay J. (2017). Benchmarking Organic Mixed Conductors for Transistors. Nat. Commun..

[12] Strakosas X., Bongo M., Owens R.M. (2015). The Organic Electrochemical Transistor for Biological Applications. J. Appl. Polym. Sci..

[13] Saftics A., Kurunczi S., Peter B., Szekacs I., Ramsden J.J., Horvath R. (2021). Data Evaluation for Surface-Sensitive Label-Free Methods to Obtain Real-Time Kinetic and Structural Information of Thin Films: A Practical Review with Related Software Packages. Adv. Colloid Interface Sci..

[14] Soltermann F., Struwe W.B., Kukura P. (2021). Label-Free Methods for Optical: In Vitro Characterization of Protein-Protein Interactions. Phys. Chem. Chem. Phys..

[15] Dzimianski J.V., Lorig-Roach N., O’Rourke S.M., Alexander D.L., Kimmey J.M., DuBois R.M. (2020). Rapid and Sensitive Detection of SARS-CoV-2 Antibodies by Biolayer Interferometry. Sci. Rep..

[16] Tang H., Lin P., Chan H.L.W., Yan F. (2011). Highly Sensitive Dopamine Biosensors Based on Organic Electrochemical Transistors. Biosens. Bioelectron..

[17] Macchia E., Romele P., Manoli K., Ghittorelli M., Magliulo M., Kovács-Vajna Z.M., Torricelli F., Torsi L. (2018). Ultra-Sensitive Protein Detection with Organic Electrochemical Transistors Printed on Plastic Substrates. Flex. Print. Electron..

[18] Berggren M., Crispin X., Fabiano S., Jonsson M.P., Simon D.T., Stavrinidou E., Tybrandt K., Zozoulenko I. (2019). Ion Electron–Coupled Functionality in Materials and Devices Based on Conjugated Polymers. Adv. Mater..

[19] Kergoat L., Piro B., Berggren M., Horowitz G., Pham M.C. (2012). Advances in Organic Transistor-Based Biosensors: From Organic Electrochemical Transistors to Electrolyte-Gated Organic Field-Effect Transistors. Anal. Bioanal. Chem..

[20] Donahue M.J., Sanchez-Sanchez A., Inal S., Qu J., Owens R.M., Mecerreyes D., Malliaras G.G., Martin D.C. (2020). Tailoring PEDOT Properties for Applications in Bioelectronics. Mater. Sci. Eng. R Rep..

[21] Hui Y., Bian C., Xia S., Tong J., Wang J. (2018). Synthesis and Electrochemical Sensing Application of Poly(3,4-Ethylenedioxythiophene)-Based Materials: A Review. Anal. Chim. Acta.

[22] Fan X., Nie W., Tsai H., Wang N., Huang H., Cheng Y., Wen R., Ma L., Yan F., Xia Y. (2019). PEDOT:PSS for Flexible and Stretchable Electronics: Modifications, Strategies, and Applications. Adv. Sci..

[23] Amirzadeh Z., Javadpour S., Shariat M.H., Knibbe R. (2018). Non-Enzymatic Glucose Sensor Based on Copper Oxide and Multi-Wall Carbon Nanotubes Using PEDOT:PSS Matrix. Synth. Met..

[24] Zhang C., Higgins T.M., Park S.H., O’Brien S.E., Long D., Coleman J.N., Nicolosi V. (2016). Highly Flexible and Transparent Solid-State Supercapacitors Based on RuO2/PEDOT:PSS Conductive Ultrathin Films. Nano Energy.

[25] Fenoy G.E., von Bilderling C., Knoll W., Azzaroni O., Marmisollé W.A. (2021). PEDOT:Tosylate-Polyamine-Based Organic Electrochemical Transistors for High-Performance Bioelectronics. Adv. Electron. Mater..

[26] Guckeisen T., Hosseinpour S., Peukert W. (2019). Isoelectric Points of Proteins at the Air/Liquid Interface and in Solution. Langmuir.

[27] Sappia L.D., Piccinini E., Marmisollé W., Santilli N., Maza E., Moya S., Battaglini F., Madrid R.E., Azzaroni O. (2017). Integration of Biorecognition Elements on PEDOT Platforms through Supramolecular Interactions. Adv. Mater. Interfaces.

[28] Terrones Y.T., Laucirica G., Cayón V.M., Fenoy G.E., Cortez M.L., Toimil-Molares M.E., Trautmann C., Mamisollé W.A., Azzaroni O. (2022). Highly Sensitive Acetylcholine Biosensing via Chemical Amplification of Enzymatic Processes in Nanochannels. Chem. Commun..

[29] Pérez-Mitta G., Peinetti A.S., Cortez M.L., Toimil-Molares M.E., Trautmann C., Azzaroni O. (2018). Highly Sensitive Biosensing with Solid-State Nanopores Displaying Enzymatically Reconfigurable Rectification Properties. Nano Lett..

[30] Pallarola D., Queralto N., Battaglini F., Azzaroni O. (2010). Supramolecular Assembly of Glucose Oxidase on Concanavalin A—Modified Gold Electrodes. Phys. Chem. Chem. Phys..

[31] Pallarola D., von Bildering C., Pietrasanta L.I., Queralto N., Knoll W., Battaglini F., Azzaroni O. (2012). Recognition-Driven Layer-by-Layer Construction of Multiprotein Assemblies on Surfaces: A Biomolecular Toolkit for Building up Chemoresponsive Bioelectrochemical Interfaces. Phys. Chem. Chem. Phys..

[32] Pallarola D., Queralto N., Knoll W., Ceolin M., Azzaroni O., Battaglini F. (2010). Redox-Active Concanavalin a: Synthesis, Characterization, and Recognition-Driven Assembly of Interfacial Architectures for Bioelectronic Applications. Langmuir.

[33] Fenoy G.E., Scotto J., Allegretto J.A., Piccinini E., Cantillo A.L., Knoll W., Azzaroni O., Marmisollé W.A. (2022). Layer-by-Layer Assembly Monitored by PEDOT-Polyamine-Based Organic Electrochemical Transistors. ACS Appl. Electron. Mater..

[34] Winther-Jensen B., West K. (2004). Vapor-Phase Polymerization of 3,4-Ethylenedioxythiophene: A Route to Highly Conducting Polymer Surface Layers. Macromolecules.

[35] Winther-Jensen B., Breiby D.W., West K. (2005). Base Inhibited Oxidative Polymerization of 3,4-Ethylenedioxythiophene with Iron(III)Tosylate. Synth. Met..

[36] Schaarschmidt A., Farah A.A., Aby A., Helmy A.S. (2009). Influence of Nonadiabatic Annealing on the Morphology and Molecular Structure of PEDOT− PSS Films. J. Phys. Chem. B.

[37] Garreau S., Louarn G., Buisson J.P., Froyer G., Lefrant S. (1999). In Situ Spectroelectrochemical Raman Studies of Poly (3, 4-Ethylenedioxythiophene)(PEDT). Macromolecules.

[38] Lawrie G., Keen I., Drew B., Chandler-Temple A., Rintoul L., Fredericks P., Grøndahl L. (2007). Interactions between Alginate and Chitosan Biopolymers Characterized Using FTIR and XPS. Biomacromolecules.

[39] Luo J., Billep D., Waechtler T., Otto T., Toader M., Gordan O., Sheremet E., Martin J., Hietschold M., Zahn D.R.T. (2013). Enhancement of the Thermoelectric Properties of PEDOT: PSS Thin Films by Post-Treatment. J. Mater. Chem. A.

[40] Scotto J., Piccinini E., von Bilderling C., Coria-Oriundo L.L., Battaglini F., Knoll W., Marmisolle W.A., Azzaroni O. (2020). Flexible Conducting Platforms Based on PEDOT and Graphite Nanosheets for Electrochemical Biosensing Applications. Appl. Surf. Sci..

[41] Rivnay J., Leleux P., Sessolo M., Khodagholy D., Hervé T., Fiocchi M., Malliaras G.G. (2013). Organic Electrochemical Transistors with Maximum Transconductance at Zero Gate Bias. Adv. Mater..

[42] Wong J.E., Zastrow H., Jaeger W., von Klitzing R. (2009). Specific Ion versus Electrostatic Effects on the Construction of Polyelectrolyte Multilayers. Langmuir.

[43] Donath E., Vardanyan I., Meyer S., Murray R.A., Moya S.E., Navoyan Z., Arakelyan V. (2018). A Typical Diffusion Monitored by Flow Cytometry: Slow Diffusion of Small Molecules in Polyelectrolyte Multilayers. Nanoscale.

[44] Sui Z., Schlenoff J.B. (2004). Phase Separations in PH-Responsive Polyelectrolyte Multilayers: Charge Extrusion versus Charge Expulsion. Langmuir.

[45] Sheldon R.A., van Pelt S. (2013). Enzyme Immobilisation in Biocatalysis: Why, What and How. Chem. Soc. Rev..

[46] Laucirica G., Marmisollé W.A., Azzaroni O. (2017). Dangerous Liaisons: Anion-Induced Protonation in Phosphate-Polyamine Interactions and Their Implications for the Charge States of Biologically Relevant Surfaces. Phys. Chem. Chem. Phys..

[47] Pazur J.H., Kleppe K. (1964). The Oxidation of Glucose and Related Compounds by Glucose Oxidase from Aspergillus Niger. Biochemistry.

[48] Sappia L.D., Piccinini E., von Binderling C., Knoll W. (2020). PEDOT-Polyamine Composite Fi Lms for Bioelectrochemical Platforms-Fl Exible and Easy to Derivatize. Mater. Sci. Eng. C.

[49] Entlicher G., Koštíř J.V., Kocourek J. (1971). Studies on Phytohemagglutinins. VIII. Isoelectric Point and Multiplicity of Purified Concanavalin A. BBA-Protein Struct..

[50] Fakih I., Durnan O., Mahvash F., Napal I., Centeno A., Zurutuza A., Yargeau V., Szkopek T. (2020). Selective Ion Sensing with High Resolution Large Area Graphene Field Effect Transistor Arrays. Nat. Commun..

[51] Kanai Y., Ohmuro-Matsuyama Y., Tanioku M., Ushiba S., Ono T., Inoue K., Kitaguchi T., Kimura M., Ueda H., Matsumoto K. (2020). Graphene Field Effect Transistor-Based Immunosensor for Ultrasensitive Noncompetitive Detection of Small Antigens. ACS Sens..

[52] Zou X., Wu J., Gu J., Shen L., Mao L. (2019). Application of Aptamers in Virus Detection and Antiviral Therapy. Front. Microbiol..

[53] Hai W., Goda T., Takeuchi H., Yamaoka S., Horiguchi Y., Matsumoto A., Miyahara Y. (2018). Human Influenza Virus Detection Using Sialyllactose-Functionalized Organic Electrochemical Transistors. Sens. Actuators B Chem..

[54] Piccinini E., Fenoy G.E., Cantillo A.L., Allegretto J.A., Scotto J., Piccinini J.M., Marmisollé W.A., Azzaroni O. (2022). Biofunctionalization of Graphene-Based FET Sensors through Heterobifunctional Nanoscaffolds: Technology Validation toward Rapid COVID-19 Diagnostics and Monitoring. Adv. Mater. Interfaces.

[55] Hinnemo M., Makaraviciute A., Ahlberg P., Olsson J., Zhang Z., Zhang S.L., Zhang Z. (2018). Bin Protein Sensing beyond the Debye Length Using Graphene Field-Effect Transistors. IEEE Sens. J..

[56] Hajian R., Balderston S., Tran T., deBoer T., Etienne J., Sandhu M., Wauford N.A., Chung J.-Y., Nokes J., Athaiya M. (2019). Detection of Unamplified Target Genes via CRISPR–Cas9 Immobilized on a Graphene Field-Effect Transistor. Nat. Biomed. Eng..

[57] Goldsmith B.R., Locascio L., Gao Y., Lerner M., Walker A., Lerner J., Kyaw J., Shue A., Afsahi S., Pan D. (2019). Digital Biosensing by Foundry-Fabricated Graphene Sensors. Sci. Rep..

[58] Kesler V., Murmann B., Soh H.T. (2020). Going beyond the Debye Length: Overcoming Charge Screening Limitations in Next-Generation Bioelectronic Sensors. ACS Nano.

[59] Gao N., Zhou W., Jiang X., Hong G., Fu T.M., Lieber C.M. (2015). General Strategy for Biodetection in High Ionic Strength Solutions Using Transistor-Based Nanoelectronic Sensors. Nano Lett..

[60] Gutiérrez-Sanz Ó., Andoy N.M., Filipiak M.S., Haustein N., Tarasov A. (2017). Direct, Label-Free, and Rapid Transistor-Based Immunodetection in Whole Serum. ACS Sens..

[61] Pappa A.M., Inal S., Roy K., Zhang Y., Pitsalidis C., Hama A., Pas J., Malliaras G.G., Owens R.M. (2017). Polyelectrolyte Layer-by-Layer Assembly on Organic Electrochemical Transistors. ACS Appl. Mater. Interfaces.

[62] Bernards D.A., Malliaras G.G. (2007). Steady-State and Transient Behavior of Organic Electrochemical Transistors. Adv. Funct. Mater..

