# Supplementary material for: PEDOT-Polyamine-Based Organic Electrochemical Transistors for Monitoring Protein Binding
Source: Biosensors (Basel). 2023 Feb 17;13(2):288. doi: 10.3390/bios13020288 (PMC9954629; doi:10.3390/bios13020288)
Supplement: Supplementary file 1 [file biosensors-13-00288-s001.zip › biosensors-2156623-supplementary.pdf]

# Supplementary Materials

# PEDOT-polyamine-based organic electrochemical transistors for monitoring protein binding

*Marjorie Montero-Jimenez*[1], *Francisco L. Amante*[1], *Gonzalo E. Fenoy*[1], *Juliana Scotto*[1]*, *Omar Azzaroni**[1], *Waldemar A. Marmisolle*[1]*

[1] Instituto de Investigaciones Fisicoquímicas Teóricas y Aplicadas (INIFTA) – Departamento de Química – Facultad de Ciencias Exactas – Universidad Nacional de La Plata (UNLP) – CONICET. 64 and 113, La Plata (1900), Buenos Aires, Argentina.

## S1. Ellipsometric measurements

An Alpha-SE Ellipsometer (J.A. Woollan) was employed to measure the thickness of PEDOT-PAH films prepared on silicon substrates with a native oxide layer. Each sample was analyzed at four different spots. Measurements were performed with an incidence angle of 70°, in the spectral range from 380 to 900 nm, using a robust sample alignment and a long acquisition time. With a Cauchy model a mean value of 1.6 nm ± 0.7 nm was obtained and the dispersion of the results is shown in **Figure S1.**

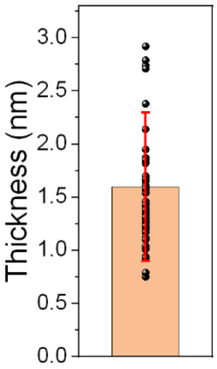


**Figure S1**. Thickness of PEDOT-PAH films prepared on Si substrates determined by ellipsometry. The synthesis conditions were the same as those employed for the construction of the OECTs.

## S2. Surface Plasmon Resonance (SPR)

The electrostatic adsorption of PSS, ConA and GOx on PEDOT-PAH surfaces were monitored by SPR on gold substrates. From the change in the minimum reflectance angle, the mass density deposited on the substrate (Γ) was estimated using Freijter's equation:

$$\Gamma = \frac{\Delta\theta kd}{dn/dc}$$

where *kd is* an instrument constant and *dn/dc* the dependency of the refraction index with the concentration. For the calculations a *dn/dc* value of 0.197 $cm^3$ $g^{-1}$ was considered for PSS and 0.177 $cm^3$ $g^{-1}$ for the proteins and a value of *kd*=1.9 x $10^{-7}$ cm was employed for the 785 nm laser and a 1.0 x $10^{-7}$ for the measurement employing the 670 nm laser.

## S3. $V_{G\ gm,\ max}$ shifts upon ConA and GOx binding on PEDOT-PAH mannosylated surfaces

OECT PEDOT-PAH channels were mannosylated using DVS as crosslinker. Then ConA was adsorbed on the surface followed by the modification with GOx. The transfer curves of the OECTs were measured in 10mM KCl and 1mM Hepes before and after each modification step. Then, the mean $V_{G\ gm,\ max}$ values obtained after each modification step were calculated and plotted in **Figure S2**.

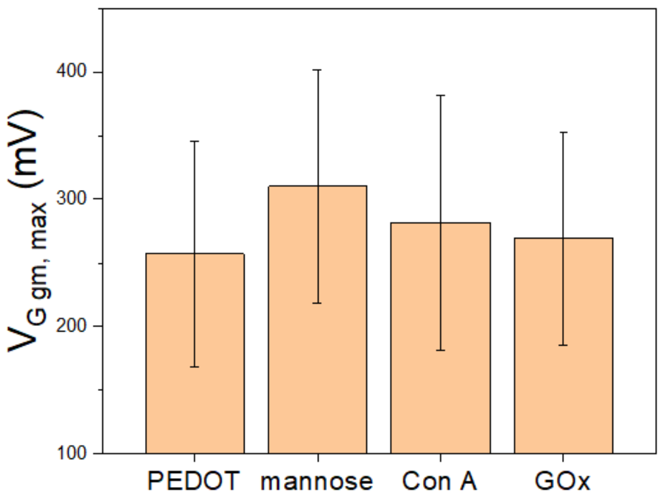


**Figure S2**. Shifts in VG $_{gm,\ max}$ due to the mannosylation, ConA and Gox adsorption (average of 4 devices).

In Figure S3, the transfer curves and the transconductance as a function of the gate potential before and after GOx adsorption on the ConA-modified surface are shown for four devices, to illustrate the reproducibility of the results.

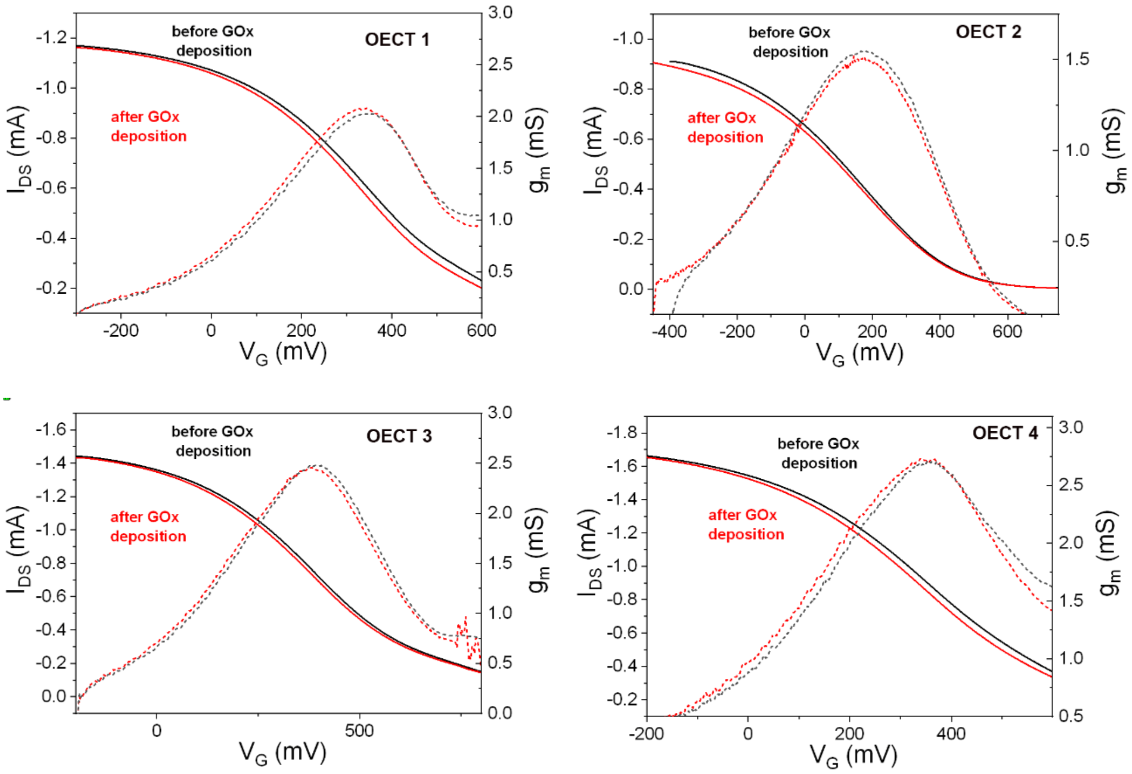


**Figure S3**. $I_{DS}$ and $g_m$ vs $V_G$ for the four devices employed in this work before (black) and after (red) the adsorption of GOx on the ConA-modified OECT. The first one (OECT 1) corresponds to the device shown in Figure 5.
